# Diagnostic performance of deep learning models using ultrasound for preoperative axillary nodal status in breast cancer: a systematic review and meta-analysis

**DOI:** 10.3389/fonc.2026.1925423

**Published:** 2026-10-23

**Authors:** Tianxin Liu, Xiaowu Yuan, Jinli Wang, Yuchen He, Yan Liu, Xiaojun Yan, Chunli Cao, Ming Chen, Wenxiao Li, Jun Li, Xinwu Cui

**Affiliations:** 1 Department of Ultrasound, the First Affiliated Hospital of Shihezi University, Shihezi, China; 2 Tongji Hospital, Affiliated to Tongji Medical College, Huazhong University of Science and Technology, Wuhan, China

**Keywords:** axillary lymph node, breast cancer, deep learning, diagnostic accuracy, meta-analysis, sentinel lymph node, ultrasound

## Abstract

**Background:**

Deep-learning (DL) models may extract nodal-risk information from breast or axillary ultrasound, but the relevant studies differ in anatomical input, preprocessing, reference standard, and validation design.

**Methods:**

We updated PubMed, Web of Science Core Collection, and Cochrane CENTRAL, with supplementary OpenAlex and Crossref searches and repeated backward and forward citation searching through 24 August 2026. Eligible studies evaluated a deep-learning model using ultrasound as the sole imaging modality for pretreatment binary axillary or sentinel nodal status in breast cancer, used surgical pathology as the reference standard, and reported an independent patient-level validation cohort with an exact or uniquely reconstructable 2 × 2 table. One cohort per study was selected using a prespecified hierarchy. Sensitivity and specificity were synthesized with a bivariate random-effects model and an HSROC curve.

**Results:**

Eleven studies comprising 1,027 validation patients were included. Pooled sensitivity was 0.75 (95% CI 0.66–0.81; 95% prediction interval 0.50–0.90) and pooled specificity was 0.89 (95% CI 0.83–0.93; prediction interval 0.66–0.97). The summary HSROC area was 0.81. The positive likelihood ratio was 6.60 (95% CI 4.29–10.42), the negative likelihood ratio was 0.29 (0.21–0.38), and the diagnostic odds ratio was 23.12 (12.89–41.50). Descriptive univariate I² values were 64.2% for sensitivity and 73.8% for specificity. Deeks’ test did not indicate funnel-plot asymmetry (P = 0.677).

**Conclusions:**

Ultrasound-based DL models showed moderate sensitivity and high specificity in selected validation cohorts. Wide prediction intervals, heterogeneous anatomical inputs and preprocessing, incompletely reported definitions of nodal positivity, and the geographic concentration of studies limit immediate clinical transportability. These models should be evaluated as adjuncts to, rather than replacements for, pathological axillary staging.

**Systematic review registration:**

https://www.crd.york.ac.uk/PROSPERO/, identifier CRD42024589342.

## 1 Introduction

Breast cancer is the most commonly diagnosed cancer worldwide (1). Axillary nodal status remains central to staging, prognosis, and locoregional treatment planning. Trials and guidelines have supported selective de-escalation of axillary surgery in defined early-stage settings (2, 3). Surgical pathology obtained by sentinel lymph node biopsy (SLNB) or axillary lymph node dissection (ALND) remains the reference standard, but both procedures are invasive and SLNB samples only selected nodes. A reliable non-invasive triage tool could refine preoperative risk assessment while preserving pathology for definitive decisions.

Preoperative axillary ultrasound and ultrasound-guided needle biopsy can identify a proportion of node-positive patients before surgery, but sensitivity varies with nodal visibility and selection criteria (4). Conventional axillary ultrasound is highly specific but misses a clinically important proportion of nodal disease. In a bivariate meta-analysis of pretherapeutic imaging, visual ultrasound assessment had a pooled sensitivity of 0.51 (95% CI 0.43-0.59) and specificity of 1.00 (0.99-1.00) (5). These values describe direct visual assessment of axillary nodes and should not be treated as a head-to-head benchmark for DL models that infer nodal status indirectly from the primary breast tumor.

Deep learning learns hierarchical image representations and has become widely used in medical image analysis (6, 7). Radiomics converts image patterns into quantitative features that may capture tumor phenotype (8). Recent DL studies have analyzed primary-tumor images, peritumoral tissue, axillary-node images, or combinations of these structures; inputs range from static B-mode images to ultrasound cine loops. They also differ in manual versus automatic segmentation and in whether deep features are combined with handcrafted radiomics or clinical variables (9–19). Pooling these studies without making the input anatomy and validation unit explicit can obscure clinically meaningful heterogeneity.

We therefore rebuilt the evidence base around a narrow diagnostic question: among patients with untreated breast cancer, how accurately do DL models using ultrasound as the sole imaging modality classify binary axillary or sentinel nodal status in an independent patient-level validation cohort, against surgical pathology? We also examined heterogeneity related to anatomical input, ultrasound format, reference standard, validation design, and model composition.

## 2 Materials and methods

### 2.1 Protocol, reporting framework, and amendment

The review protocol was registered in PROSPERO (CRD42024589342). The revision was reported in accordance with PRISMA 2020 and PRISMA-DTA (20, 21). Before re-analysis, a dated amendment (14 August 2026) narrowed the quantitative target to DL ultrasound models with independent patient-level validation and reconstructable 2 × 2 data. The original invasive-ductal-carcinoma-only framing was removed because most eligible cohorts enrolled mixed breast-cancer histologies and did not report separable histology-specific diagnostic tables.

### 2.2 Search strategy

For this revision, PubMed was updated through 23 August 2026, Web of Science Core Collection and Cochrane CENTRAL were searched on 21 August 2026, and backward and forward citation searching was repeated. OpenAlex and Crossref were searched on 24 August 2026 as supplementary public sources. Embase could not be updated because institutional access was unavailable; the public supplementary searches do not replace Embase. Search concepts combined breast neoplasms, axillary or sentinel lymph nodes, ultrasound, and artificial intelligence, deep learning, or radiomics. Complete source-specific strategies and the search log are provided in **Appendix 1** and the screening database.

### 2.3 Eligibility criteria

Studies were eligible when they: (1) enrolled patients with breast cancer before neoadjuvant systemic therapy; (2) evaluated a DL model for binary ALN or SLN metastasis; (3) used ultrasound as the sole imaging modality, although clinical variables or handcrafted radiomics derived from the same ultrasound examination could be added; (4) used SLNB, ALND, or surgical histopathology as the reference standard; (5) reported an independent patient-level validation cohort; and (6) provided exact or uniquely reconstructable TP, FP, TN, and FN counts. Models using primary-tumor images, axillary-node images, or both were eligible but were classified separately.

We excluded traditional machine-learning-only models; models requiring MRI, CT, pathology slides, photoacoustic imaging, or other non-ultrasound imaging; post-neoadjuvant residual-disease models; nodal-burden-only or non-sentinel-node-only targets; node- or image-level analyses that could not be converted to patient level; cross-validation summaries without an independent holdout; and reports with internally incompatible denominators or performance measures.

### 2.4 Study selection and data extraction

Two-pass screening was used: an automated high-sensitivity first pass followed by manual full-text adjudication against the revised criteria. For each study, we extracted design, setting, selected validation cohort, ultrasound input, anatomical structure, ROI/preprocessing approach, model type, pathological reference standard, reported definition of nodal positivity, and TP/FP/TN/FN. Every reconstructed table was checked by recomputing sensitivity, specificity, cohort size, and disease prevalence.

To avoid optimistic multiplicity, one cohort and one model per study were selected. The cohort hierarchy was external validation, prospective or temporal validation, and then an independent internal holdout. The final model prespecified by the authors was used; when multiple external cohorts were available, the largest eligible external cohort was selected. We did not choose the threshold or model with the highest Youden index. If rounded measures mapped to more than one integer table, the study was excluded from four-cell pooling.

### 2.5 Risk of bias and applicability

Risk of bias and applicability were assessed with QUADAS-2 (22). Patient selection, index-test conduct/interpretation, reference standard, and flow/timing were judged as low, high, or unclear risk. Applicability was evaluated for population selection, model input, and target condition. Particular attention was paid to restricted populations, threshold selection, and whether the model assessed primary-tumor risk versus direct axillary-node morphology.

### 2.6 Statistical analysis

Sensitivity and specificity were synthesized jointly on the logit scale using a bivariate random-effects model (23), with the corresponding HSROC representation (24). Zero cells received a 0.5 continuity correction only for study-level logit calculations and display. We report pooled sensitivity and specificity with 95% confidence intervals and 95% prediction intervals, the summary ROC area, positive and negative likelihood ratios, diagnostic odds ratio, and Fagan post-test probabilities.

Between-study heterogeneity was represented primarily by random-effects variances, the correlation between logit sensitivity and logit specificity, and prediction intervals. Cochran Q and univariate I² for sensitivity and specificity were also computed to address reviewer requests; because these measures ignore the intrinsic threshold-dependent correlation, they are presented as descriptive rather than primary heterogeneity statistics. Deeks’ funnel-plot asymmetry test was used because at least 10 studies were available (25). Subgroups with at least four studies were estimated exploratorily; no causal or between-subgroup inference was made.

The primary model was fit independently by maximum likelihood in Python and by restricted maximum likelihood with the metafor package in R (26). The R estimates were used for reported pooled sensitivity and specificity; all other derived quantities and figures came from the concordant Python fit. Analyses were executed under Slurm on 14 August 2026.

## 3 Results

### 3.1 Search results

The completed searches identified 531 source records (PubMed, 161; Web of Science, 170; Cochrane CENTRAL, 3; OpenAlex supplementary search, 115; Crossref supplementary search, 82). After removal of 295 duplicates, 236 records were screened and 200 were excluded from title and abstract. Citation searching identified 1 additional record, which was excluded after title and abstract screening. We sought 36 reports in total; all 36 were assessed in full text. 25 reports were excluded after full-text assessment. 11 studies were included in the quantitative synthesis (**Figure 1**).

**Figure 1:**
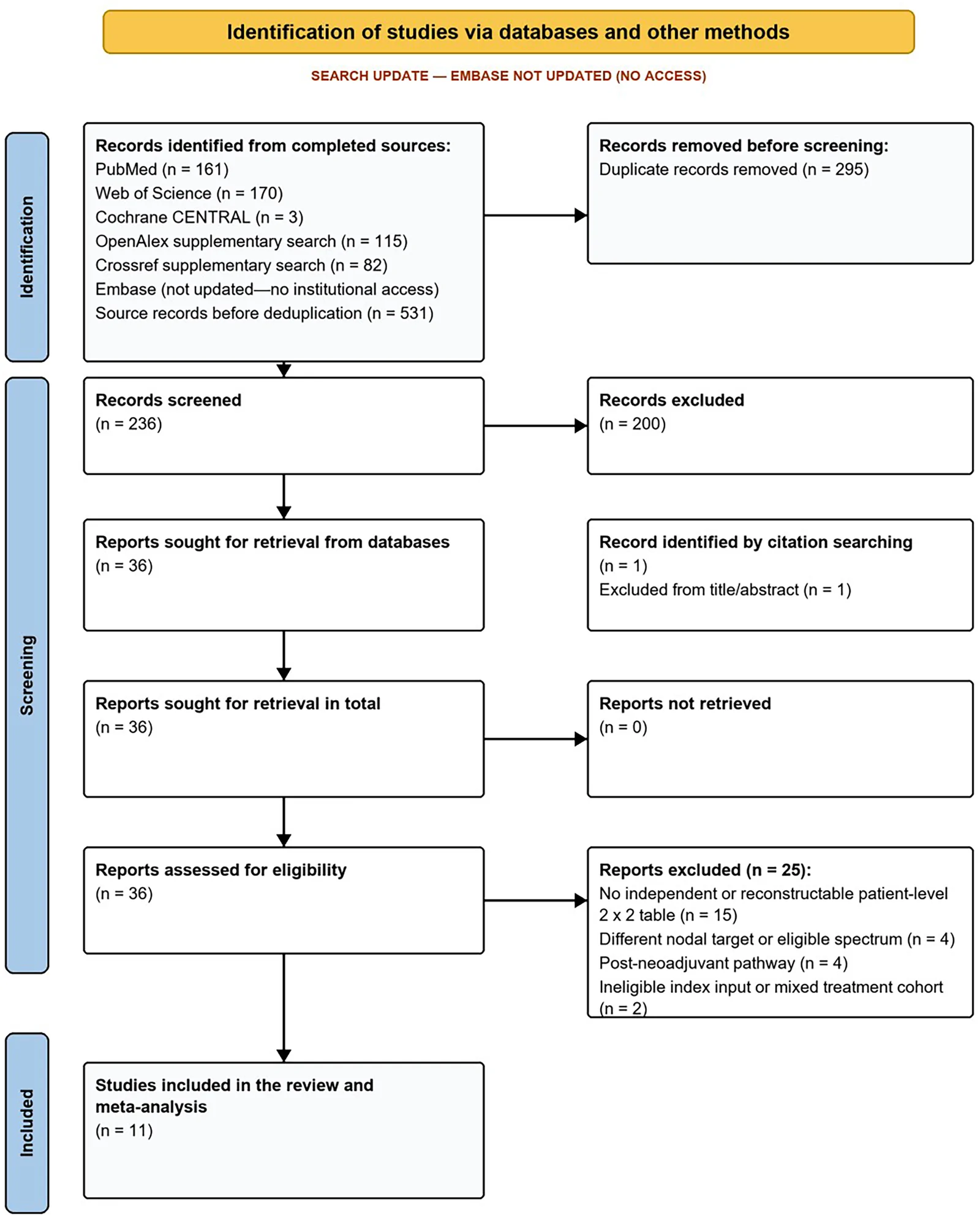
PRISMA flow diagram for the revised evidence base.

All 36 reports sought for retrieval were obtained and assessed in full text.

### 3.2 Study characteristics

The 11 selected validation cohorts included 1,027 patients and were published from 2020 to 2026 (9–19). All studies were conducted in China. Eight selected cohorts were external, prospective, or temporal validations, whereas three were independent internal holdouts. Eight models used only primary-tumor ultrasound, three incorporated axillary-node images, ten used static B-mode input, and one used ultrasound video. **Table 1** separates anatomical input, preprocessing, model composition, reference standard, and nodal-positivity reporting. The selected validation cohorts and patient-level diagnostic data used in the primary bivariate meta-analysis are summarized in **Table 2**.

**Table 1:** Study characteristics, anatomical inputs, preprocessing, and reference standards.

Study	Design/setting	US input and anatomy	ROI/preprocessing	Model	Reference standard	Nodal positivity
Sun 2020 (9)	Prospective-retrospective single center; China	B-mode primary-tumor images; ALN status	Manual intra- and peritumoral ROIs	Image-only CNN combined intratumoral and peritumoral model	SLNB or ALND	Pathology-defined N+; micrometastasis/ITC threshold not consistently reported
Wei 2023 (10)	Retrospective single center; China	B-mode primary-tumor images; ALN status	Semiautomatic tumor segmentation	DLR combined with radiomics and clinical parameters	ALND	Pathology-defined N+; micrometastasis/ITC threshold not consistently reported
Li 2023 (11)	Retrospective multicenter; China	Dynamic B-mode primary-tumor ultrasound video; ALN status	Manual ROI/key-frame video processing	TIN video model	Surgical pathology	Pathology-defined N+; micrometastasis/ITC threshold not consistently reported
Qian 2024 (12)	Retrospective single center; China	B-mode primary-tumor images; ALN status	Manual primary-tumor ROI	Integrated deep-learning-radiomics nomogram	SLNB or ALND	Pathology-defined N+; micrometastasis/ITC threshold not consistently reported
Liu 2024 (13)	Retrospective multicenter; China	B-mode primary-tumor image plus preoperative variables; ALN status	Primary-tumor ROI; deep and handcrafted feature extraction	Deep-learning radiomics nomogram	SLNB or ALND	Pathology-defined N+; micrometastasis/ITC threshold not consistently reported
Wang 2024 (14)	Retrospective single center; China	Axillary B-mode ultrasound images; Target ALN status	Automatic axillary-node segmentation	DeepLabV3+ segmentation plus ResNet-101 classifier	SLNB or ALND	Pathology-defined N+; micrometastasis/ITC threshold not consistently reported
Zhang 2025 (15)	Prospective-retrospective multicenter; China	B-mode primary-tumor and ipsilateral ALN images; ALN status	Manual tumor and node ROIs	Clinic-radiomics model using deep features	SLNB or ALND	Pathology-defined N+; micrometastasis/ITC threshold not consistently reported
Li 2025 (16)	Retrospective multicenter; China	B-mode primary-tumor and ALN images; ALN status	Manual tumor/node annotations; multi-instance aggregation	Two-stage MTL-MIL framework with SegFormer	Histopathology	Pathology-defined N+; micrometastasis/ITC threshold not consistently reported
Agyekum 2025 (17)	Retrospective multicenter; China	B-mode primary-tumor image; ALN status	Primary-tumor ROI; graph construction from deep features	GCN using ultrasound-derived deep features at threshold 0.95	SLNB or ALND	Pathology-defined N+; micrometastasis/ITC threshold not consistently reported
Liu 2025 (18)	Retrospective two center; China	B-mode primary-tumor image; SLN status	Automatic tumor segmentation after reference annotation	Fully automated combined DL model	SLNB or ALND	Pathology-defined N+; micrometastasis/ITC threshold not consistently reported
Zhang 2026 (19)	Retrospective single center; China	B-mode primary-tumor image; ALN status	Preprocessed primary-tumor ROI; attention-guided features	CBAM-DenseNet201 radiomics-clinical fusion model	Pathology	Pathology-defined N+; micrometastasis/ITC threshold not consistently reported

ALN, axillary lymph node; DL, deep learning; GCN, graph convolutional network; ITC, isolated tumor cells; ROI, region of interest; SLN, sentinel lymph node. “Not consistently reported” indicates that deposit-size handling could not be harmonized from the report.

**Table 2:** Patient-level diagnostic data used in the primary bivariate meta-analysis.

Study	Selected validation	n	TP	FP	TN	FN	Sensitivity	Specificity	AUC
Sun 2020 (9)	Prospective cohort	16	4	1	9	2	0.67	0.90	0.950
Wei 2023 (10)	Independent test cohort	179	38	10	122	9	0.81	0.92	0.920
Li 2023 (11)	External test set B	49	6	3	35	5	0.55	0.92	0.773
Qian 2024 (12)	Temporal validation cohort	75	20	8	42	5	0.80	0.84	0.906
Liu 2024 (13)	External validation cohort 1	112	39	8	59	6	0.87	0.88	0.914
Wang 2024 (14)	Independent test set	27	4	0	20	3	0.57	1.00	0.799
Zhang 2025 (15)	Prospective test set	107	41	4	49	13	0.76	0.92	0.950
Li 2025 (16)	External test cohort	140	80	2	44	14	0.85	0.96	0.918
Agyekum 2025 (17)	Validation cohort 1	112	22	4	63	23	0.49	0.94	0.880
Liu 2025 (18)	External test set	113	42	21	36	14	0.75	0.63	0.763
Zhang 2026 (19)	Independent test group	97	37	9	41	10	0.79	0.82	0.834

One validation cohort per study was selected using the prespecified hierarchy. Counts were exact or uniquely reconstructed and independently checked against reported cohort totals and accuracy measures.

### 3.3 Risk of bias and applicability

Reference-standard and flow/timing domains were generally low risk because surgical pathology was used and validation data were linked at patient level. Patient selection was frequently unclear in retrospective cohorts. Qian et al. enrolled only patients aged 75 years or older, and Wang et al. selected patients with suspicious axillary ultrasound findings; both were judged to raise applicability concerns. Index-test risk was usually unclear because prespecification of classification thresholds was incompletely described; Agyekum et al. was judged high risk for threshold-related concerns. The study-level assessment is shown in **Figure 2**.

**Figure 2:**
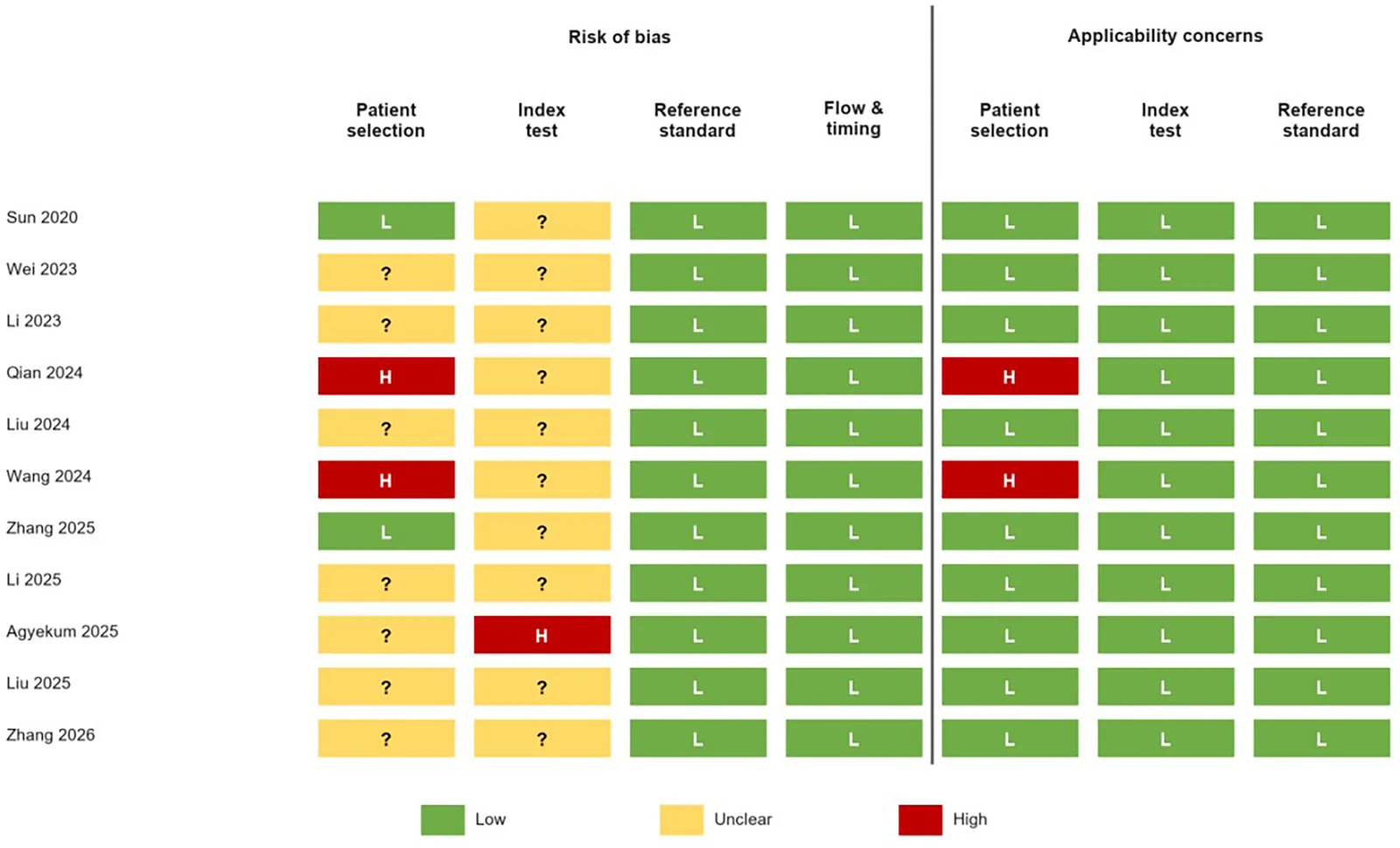
QUADAS-2 study-level risk-of-bias and applicability assessment.

### 3.4 Primary diagnostic accuracy

The bivariate random-effects model yielded a pooled sensitivity of 0.75 (95% CI 0.66–0.81) and specificity of 0.89 (95% CI 0.83–0.93). The 95% prediction intervals were 0.50–0.90 for sensitivity and 0.66–0.97 for specificity, demonstrating materially wider uncertainty for a future setting than the confidence intervals around the means. Forest plots are shown in **Figures 3** and **4**.

**Figure 3:**
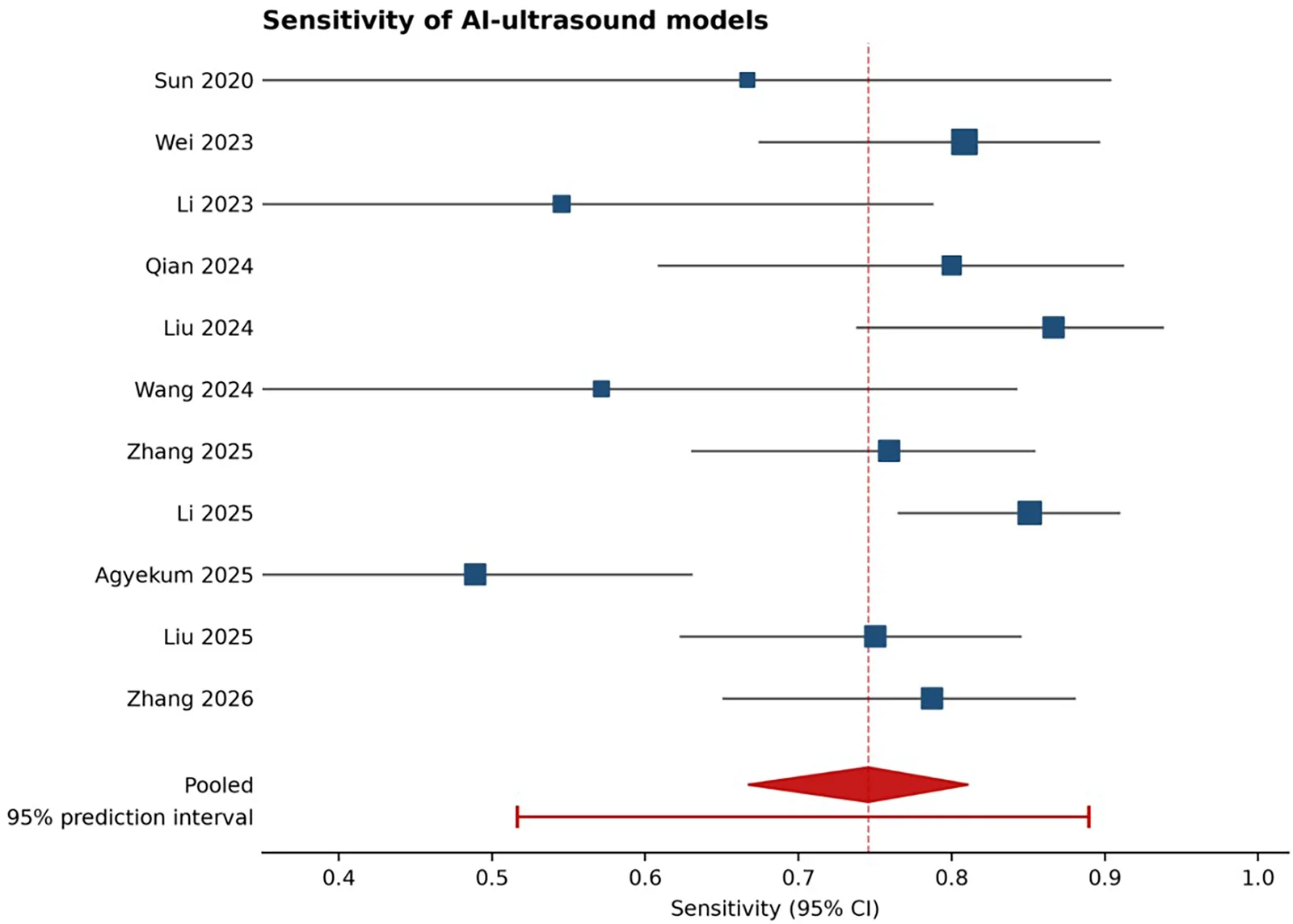
Forest plot of patient-level sensitivity.

**Figure 4:**
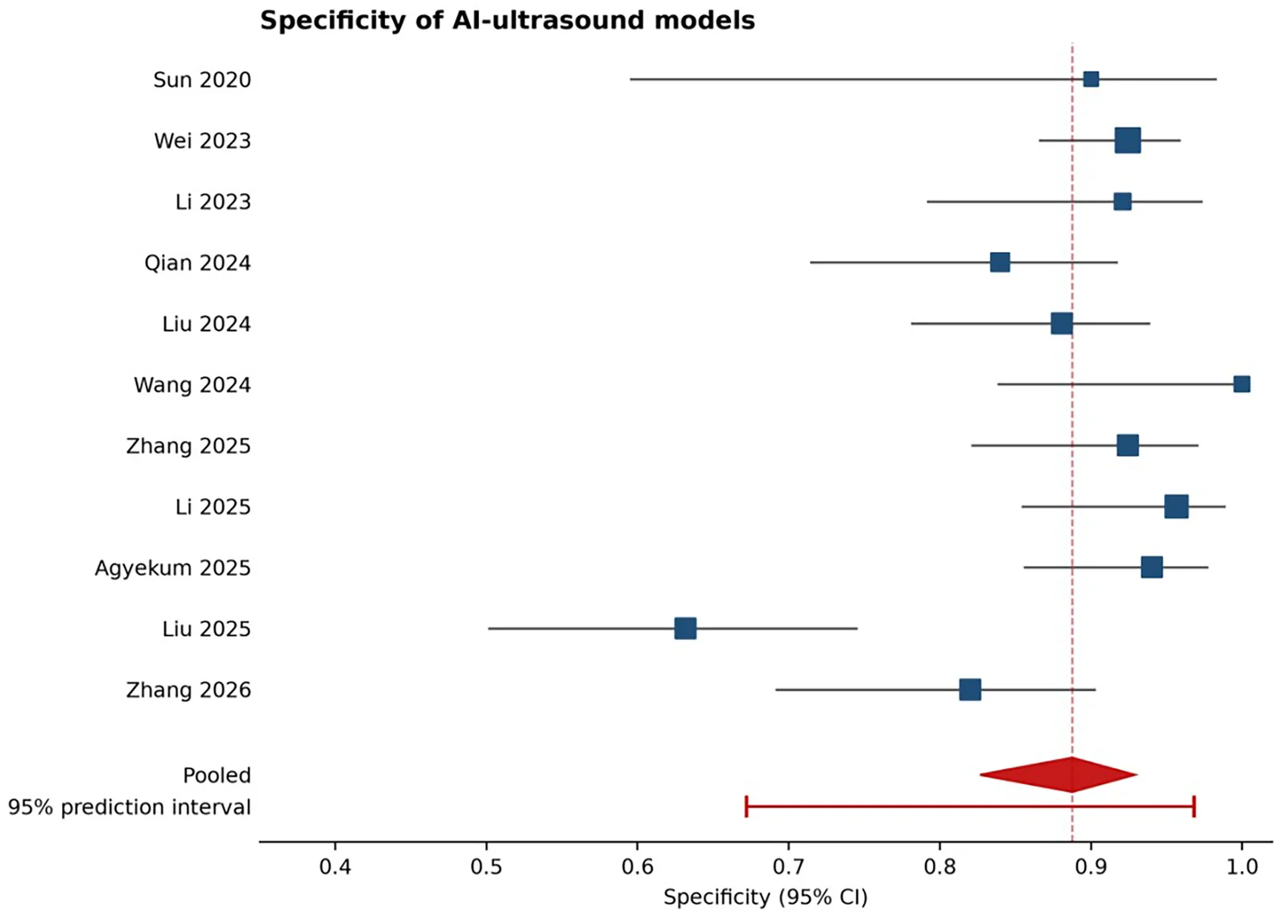
Forest plot of patient-level specificity.

The summary HSROC area was 0.81 (**Figure 5**). The positive likelihood ratio was 6.60 (95% CI 4.29–10.42), negative likelihood ratio 0.29 (0.21–0.38), and diagnostic odds ratio 23.12 (12.89–41.50). At pretest probabilities of 20% and 30%, a positive model result increased the corresponding post-test probabilities to 62.3% and 73.9%, whereas a negative result reduced them to 6.7% and 11.0%, respectively.

**Figure 5:**
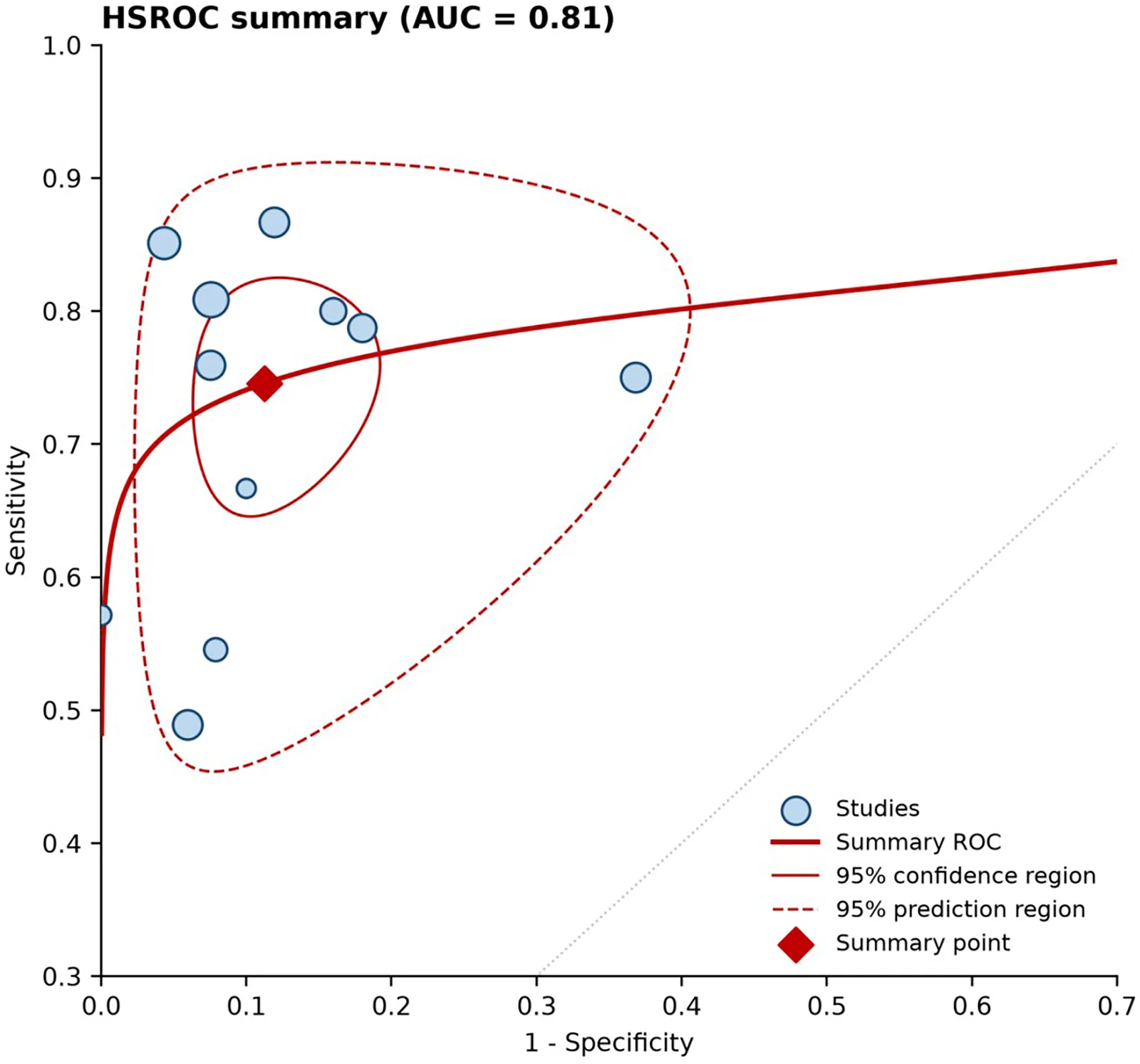
Hierarchical summary receiver-operating-characteristic curve with confidence and prediction regions.

### 3.5 Heterogeneity, subgroups, and reporting bias

The random-effects standard deviations were 0.51 for logit sensitivity and 0.68 for logit specificity in the REML validation fit; the estimated correlation was −0.26. Descriptive univariate heterogeneity was I² = 64.2% for sensitivity (Q = 27.94, 10 df, P = 0.0018) and I² = 73.8% for specificity (Q = 38.24, 10 df, P < 0.001). These values support substantial dispersion but do not capture the bivariate threshold structure.

Exploratory subgroup estimates are shown in **Table 3**. Primary-tumor-only models had sensitivity 0.73 (95% CI 0.63–0.81) and specificity 0.87 (0.79–0.92). Models using explicit SLNB/ALND reference standards had sensitivity 0.73 (0.63–0.81) and specificity 0.89 (0.80–0.94). Generalizability-focused validations had sensitivity 0.74 (0.64–0.82) and specificity 0.88 (0.80–0.93). DL-only models were more specific but less sensitive than hybrid models; because subgroups were small and no formal interaction test was prespecified, these patterns are hypothesis-generating. The Deeks' funnel plot used to assess small-study effects is shown in **Figure 6**.

**Table 3:** Exploratory bivariate subgroup estimates.

Subgroup	k	Sensitivity (95% CI)	Specificity (95% CI)
Primary-tumor-only input	8	0.73 (0.63–0.81)	0.87 (0.79–0.92)
Static B-mode input	10	0.76 (0.68–0.82)	0.88 (0.82–0.93)
Explicit SLNB/ALND reference	8	0.73 (0.63–0.81)	0.89 (0.80–0.94)
Generalizability-focused validation	8	0.74 (0.64–0.82)	0.88 (0.80–0.93)
DL-only final model	5	0.65 (0.47–0.79)	0.94 (0.89–0.97)
Hybrid DL-radiomics/clinical	6	0.79 (0.74–0.84)	0.85 (0.76–0.91)
Published through 2024	6	0.76 (0.65–0.85)	0.90 (0.85–0.94)
Published 2025-2026	5	0.74 (0.63–0.83)	0.87 (0.75–0.94)

All subgroup estimates are exploratory. No formal between-subgroup interaction test was performed.

**Figure 6:**
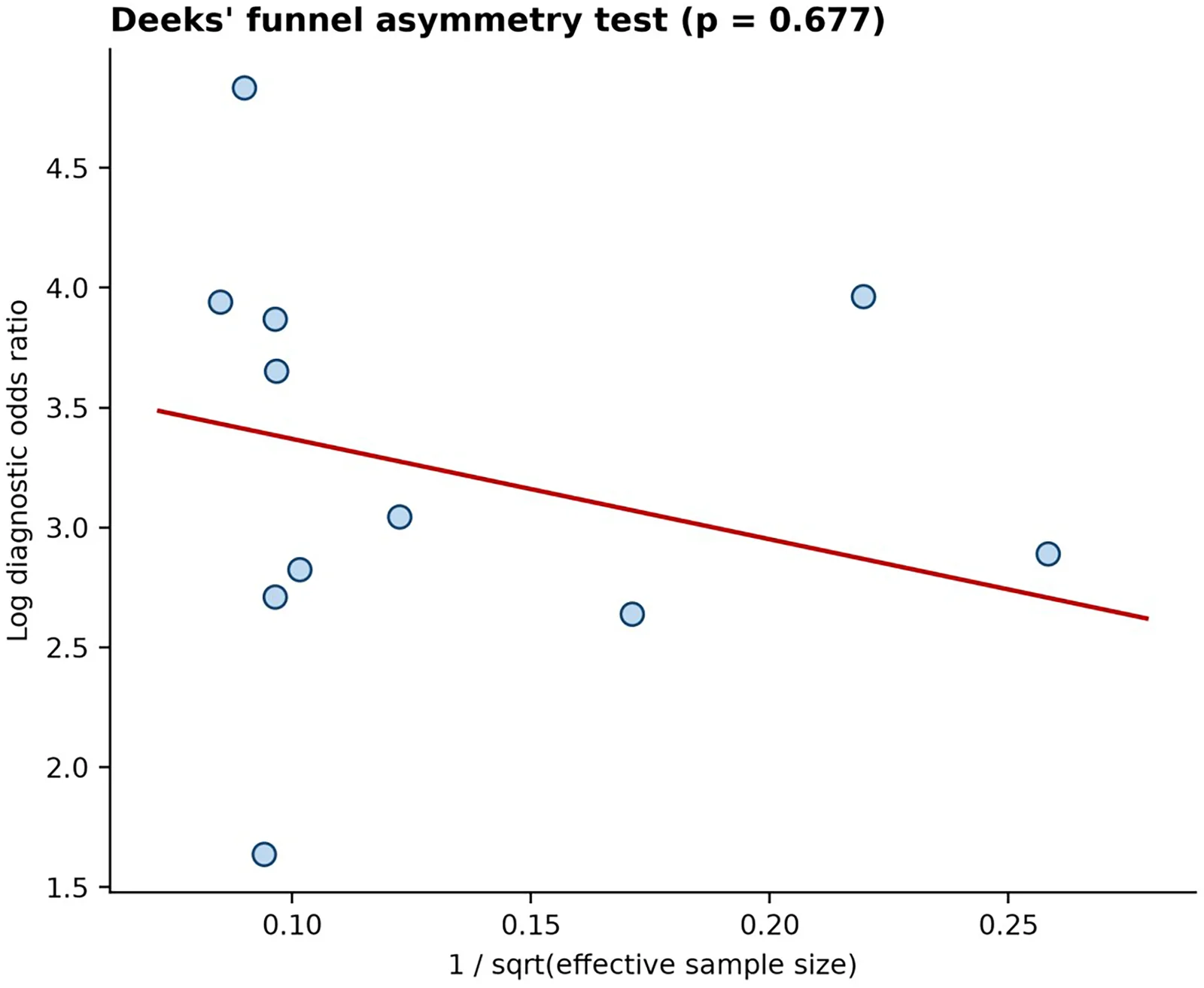
Deeks’ funnel plot for small-study effects.

Deeks’ regression test did not indicate asymmetry (P = 0.677). With only 11 studies, absence of statistical evidence should not be interpreted as proof that reporting bias is absent.

## 4 Discussion

### 4.1 Principal findings

In 11 independently evaluated cohorts, ultrasound-based DL models showed moderate sensitivity and high specificity for pretreatment binary nodal status. The summary estimates suggest that a positive result may provide useful rule-in information, whereas the negative likelihood ratio of 0.29 is not sufficiently low to replace pathological staging when missing nodal disease would change management. Wide prediction intervals indicate that performance in a new center could differ substantially from the pooled mean.

### 4.2 Interpretation relative to conventional ultrasound

The conventional-ultrasound estimate cited in the Introduction reflects radiologist assessment of axillary-node morphology (5). It is contextual rather than a direct comparator. Most DL models in this review analyzed the primary breast tumor and inferred the probability of nodal metastasis, whereas direct conventional axillary ultrasound assesses the nodes themselves. Differences in anatomical target, patient spectrum, threshold, and reference standard preclude claims of superiority without within-patient head-to-head studies.

### 4.3 Anatomical input and preprocessing

The revised tables make explicit that eight models used primary-tumor-only input and three included axillary-node images. These strategies answer related but non-equivalent questions: tumor-based models estimate metastatic propensity indirectly, while node-based models attempt direct lesion classification. ROI generation also ranged from manual tumor or node annotation to automatic segmentation and video/key-frame processing. The primary-tumor-only subgroup remained broadly consistent with the overall estimate, but the small number of node-input studies prevented stable separate pooling. Because radiomic features are sensitive to segmentation, discretization, and preprocessing, standardized definitions such as those of the Image Biomarker Standardization Initiative may improve reproducibility (27). Future reports should provide image-unit and patient-unit flow, blinded threshold selection, complete preprocessing details, and external calibration.

### 4.4 Reference-standard and target-condition heterogeneity

SLNB and ALND are both pathological standards, but they sample different extents of the axilla. Generic descriptions of “surgical pathology” were used in three studies, and explicit handling of isolated tumor cells and micrometastases was often missing. Consequently, the positive class may not have been identical across cohorts. Restricting a subgroup to reports explicitly using SLNB and/or ALND produced similar mean estimates, but sparse data and incomplete deposit-size definitions prevent excluding clinically important heterogeneity.

### 4.5 Clinical and research implications

At a 20% pretest probability, the pooled likelihood ratios translate to approximately 62% post-test probability after a positive result and 7% after a negative result. These values may support triage or second-reader use, but the appropriate action threshold depends on whether the intended pathway is to select needle sampling, SLNB, or observation. Prospective multicenter impact studies should prespecify the clinical role, lock the model and threshold before external testing, compare the model directly with radiologist-performed axillary ultrasound under the same reference standard, and report design, data partitioning, reference standards, and performance estimates in line with current medical-imaging AI guidance such as CLAIM (28).

### 4.6 Strengths and limitations

This revision has four strengths: a clinically explicit target question; one prespecified validation cohort per study; verified patient-level four-cell data rather than copied summary estimates; and independent Python and R implementations of the bivariate model. It also separates anatomy, preprocessing, reference standard, and validation design rather than treating all “ultrasound AI” studies as interchangeable.

Limitations remain. All included studies were from China, most were retrospective, and some evaluated selected populations. Threshold prespecification and nodal deposit-size definitions were incompletely reported. Embase could not be updated because institutional access was unavailable. OpenAlex and Crossref were searched as supplementary public sources, but these searches do not replace Embase. Sparse subgroups precluded reliable meta-regression, and Deeks’ test had limited power. The analysis estimates diagnostic accuracy, not patient outcomes or the safety of omitting surgery.

## 5 Conclusions

Ultrasound-based DL models achieved pooled sensitivity of 0.75 and specificity of 0.89 in 1,027 independently evaluated patients. The results support potential use as an adjunctive risk-stratification or second-reader tool, but wide prediction intervals, heterogeneous anatomy and preprocessing, incomplete nodal-positivity definitions, and limited geographic validation preclude replacing pathological axillary staging. Prospective, threshold-locked, multicenter head-to-head studies are required before clinical implementation.

## Data Availability

The original contributions presented in the study are included in the article/supplementary material. Further inquiries can be directed to the corresponding author.

## Appendix

### Appendix 1. Reproducible search strategies

PubMed: (“Breast Neoplasms”[Mesh] OR breast cancer[Title/Abstract] OR breast carcinoma[Title/Abstract]) AND (“Lymphatic Metastasis”[Mesh] OR axillary lymph node*[Title/Abstract] OR sentinel lymph node*[Title/Abstract] OR nodal status[Title/Abstract]) AND (“Ultrasonography”[Mesh] OR ultrasound[Title/Abstract] OR ultrasonograph*[Title/Abstract] OR sonograph*[Title/Abstract] OR elastograph*[Title/Abstract]) AND (“Artificial Intelligence”[Mesh] OR artificial intelligence[Title/Abstract] OR deep learning[Title/Abstract] OR neural network*[Title/Abstract] OR machine learning[Title/Abstract] OR radiomics[Title/Abstract]). Web of Science Core Collection, Topic: (“breast cancer*” OR “breast neoplasm*” OR “breast carcinoma*”) AND (“axillary lymph node*” OR “sentinel lymph node*” OR axilla*) AND (ultrasonograph* OR ultrasound OR sonograph*) AND (“deep learning” OR “artificial intelligence” OR “neural network*” OR “convolutional neural network*” OR radiomics). Cochrane CENTRAL, Title Abstract Keyword: (“breast cancer” OR “breast neoplasm” OR “breast carcinoma”) AND (“axillary lymph node” OR “sentinel lymph node” OR axilla*) AND (ultrasonograph* OR ultrasound OR sonograph*) AND (“deep learning” OR “artificial intelligence” OR “neural network” OR “convolutional neural network” OR radiomics). OpenAlex and Crossref supplementary searches: breast cancer axillary lymph node ultrasound deep learning; breast cancer sentinel lymph node ultrasound artificial intelligence; breast cancer nodal status ultrasonography radiomics; breast carcinoma axillary lymph node ultrasound machine learning; breast cancer axillary ultrasound transformer neural network. Records had to contain terms from all four concept groups before deduplication. Planned Embase strategy, not executed because institutional access was unavailable: (‘breast tumor’/exp OR ‘breast cancer’:ti,ab OR ‘breast neoplasm’:ti,ab OR ‘breast carcinoma’:ti,ab) AND (‘lymph node metastasis’/exp OR ‘axillary lymph node’:ti,ab OR ‘axillary lymph nodes’:ti,ab OR ‘sentinel lymph node’:ti,ab OR ‘sentinel lymph nodes’:ti,ab OR axilla*:ti,ab OR ‘nodal status’:ti,ab) AND (‘ultrasonography’/exp OR ultrasound:ti,ab OR ultrasonograph*:ti,ab OR sonograph*:ti,ab OR elastograph*:ti,ab) AND (‘artificial intelligence’/exp OR ‘machine learning’/exp OR ‘artificial intelligence’:ti,ab OR ‘deep learning’:ti,ab OR ‘neural network’:ti,ab OR ‘neural networks’:ti,ab OR ‘convolutional neural network’:ti,ab OR ‘convolutional neural networks’:ti,ab OR radiomics:ti,ab).

Search update: PubMed 23 August 2026 (161 records); Web of Science and CENTRAL 21 August 2026 (170 and 3 records); OpenAlex and Crossref supplementary searches 24 August 2026 (115 and 82 unique source records). Embase was not updated because institutional access was unavailable.

## References

[1] Sung H Ferlay J Siegel RL Laversanne M Soerjomataram I Jemal A . Global cancer statistics 2020: GLOBOCAN estimates of incidence and mortality worldwide for 36 cancers in 185 countries. CA Cancer J Clin. (2021) 71:209–49. doi: 10.3322/caac.21660 33538338

[2] Giuliano AE Ballman KV McCall L Beitsch PD Brennan MB Kelemen PR . Effect of axillary dissection vs no axillary dissection on 10-year overall survival among women with invasive breast cancer and sentinel node metastasis: the ACOSOG Z0011 (Alliance) randomized clinical trial. JAMA. (2017) 318:918–26. doi: 10.1001/jama.2017.11470 28898379 PMC5672806

[3] Park KU Somerfield MR Anne N Brackstone M Conlin AK Lima Couto H . Sentinel lymph node biopsy in early-stage breast cancer: ASCO guideline update. J Clin Oncol. (2025) 43:1720–41. doi: 10.1200/JCO-25-00099 40209128

[4] Houssami N Ciatto S Turner RM Cody H Macaskill P . Preoperative ultrasound-guided needle biopsy of axillary nodes in invasive breast cancer: meta-analysis of its accuracy and utility in staging the axilla. Ann Surg. (2011) 254:243–51. doi: 10.1097/SLA.0b013e31821f1564 21597359

[5] Le Boulc'h M Gilhodes J Steinmeyer Z Molière S Mathelin C . Pretherapeutic imaging for axillary staging in breast cancer: a systematic review and meta-analysis of ultrasound, MRI and FDG PET. J Clin Med. (2021) 10:1543. doi: 10.3390/jcm10071543 33917590 PMC8038849

[6] LeCun Y Bengio Y Hinton G . Deep learning. Nature. (2015) 521:436–44. doi: 10.1038/nature14539 26017442

[7] Litjens G Kooi T Bejnordi BE Setio AAA Ciompi F Ghafoorian M . A survey on deep learning in medical image analysis. Med Image Anal. (2017) 42:60–88. doi: 10.1016/j.media.2017.07.005 28778026

[8] Lambin P Rios-Velazquez E Leijenaar R Carvalho S van Stiphout RGPM Granton P . Radiomics: extracting more information from medical images using advanced feature analysis. Eur J Cancer. (2012) 48:441–6. doi: 10.1016/j.ejca.2011.11.036 22257792 PMC4533986

[9] Sun Q Lin X Zhao Y Li L Yan K Liang D . Deep learning vs. radiomics for predicting axillary lymph node metastasis of breast cancer using ultrasound images: don't forget the peritumoral region. Front Oncol. (2020) 10:53. doi: 10.3389/fonc.2020.00053 32083007 PMC7006026

[10] Wei W Ma Q Feng H Wei T Jiang F Fan L . Deep learning radiomics for prediction of axillary lymph node metastasis in patients with clinical stage T1-2 breast cancer. Quant Imaging Med Surg. (2023) 13:4995–5011. doi: 10.21037/qims-22-1257 37581073 PMC10423344

[11] Li WB Du ZC Liu YJ Gao JX Wang JG Dai Q . Prediction of axillary lymph node metastasis in early breast cancer patients with ultrasonic videos based deep learning. Front Oncol. (2023) 13:1219838. doi: 10.3389/fonc.2023.1219838 37719009 PMC10503049

[12] Qian L Liu X Zhou S Zhi W Zhang K Li H . A cutting-edge deep learning-and-radiomics-based ultrasound nomogram for precise prediction of axillary lymph node metastasis in breast cancer patients ≥ 75 years. Front Endocrinol (Lausanne). (2024) 15:1323452. doi: 10.3389/fendo.2024.1323452 39072273 PMC11272464

[13] Liu H Zou L Xu N Shen H Zhang Y Wan P . Deep learning radiomics based prediction of axillary lymph node metastasis in breast cancer. NPJ Breast Cancer. (2024) 10:22. doi: 10.1038/s41523-024-00628-4 38472210 PMC10933422

[14] Wang X Nie L Zhu Q Zuo Z Liu G Sun Q . Artificial intelligence assisted ultrasound for the non-invasive prediction of axillary lymph node metastasis in breast cancer. BMC Cancer. (2024) 24:910. doi: 10.1186/s12885-024-12619-6 39075447 PMC11285453

[15] Zhang D Zhou W Lu WW Qin XC Zhang XY Luo YH . Ultrasound-based deep learning radiomics for enhanced axillary lymph node metastasis assessment: a multicenter study. Oncologist. (2025) 30:oyaf090. doi: 10.1093/oncolo/oyaf090 40349137 PMC12065944

[16] Li Y Chen Z Ding Z Mei D Liu Z Wang J . Applying a multi-task and multi-instance framework to predict axillary lymph node metastases in breast cancer. NPJ Precis Oncol. (2025) 9:195. doi: 10.1038/s41698-025-00971-0 40533499 PMC12177086

[17] Agyekum EA Kong W Agyekum DN Issaka E Wang X Ren YZ . Ultrasound derived deep learning features for predicting axillary lymph node metastasis in breast cancer using graph convolutional networks in a multicenter study. Sci Rep. (2025) 15:27796. doi: 10.1038/s41598-025-13086-0 40738938 PMC12311213

[18] Liu T Zhao G Wei W Zhang Q Wu J Chen X . Development of fully automated deep-learning-based approach for prediction of sentinel lymph node metastasis in breast cancer patients using ultrasound imaging. Front Oncol. (2025) 15:1592521. doi: 10.3389/fonc.2025.1592521 40951361 PMC12423051

[19] Zhang H Zhao T Zhang S Wang ZY Cao NN Ding JY . Ultrasound-based attention-guided deep learning combined with radiomics to predict axillary lymph node metastasis in breast cancer. BMC Med Imaging. (2026) 26:129. doi: 10.1186/s12880-026-02208-3 41664021 PMC12983787

[20] Page MJ McKenzie JE Bossuyt PM Boutron I Hoffmann TC Mulrow CD . The PRISMA 2020 statement: an updated guideline for reporting systematic reviews. BMJ. (2021) 372:n71. doi: 10.1136/bmj.n71 33782057 PMC8005924

[21] McInnes MDF Moher D Thombs BD McGrath TA Bossuyt PM PRISMA-DTA Group . Preferred reporting items for a systematic review and meta-analysis of diagnostic test accuracy studies: the PRISMA-DTA statement. JAMA. (2018) 319:388–96. doi: 10.1001/jama.2017.19163 29362800

[22] Whiting PF Rutjes AWS Westwood ME Mallett S Deeks JJ Reitsma JB . QUADAS-2: a revised tool for the quality assessment of diagnostic accuracy studies. Ann Intern Med. (2011) 155:529–36. doi: 10.7326/0003-4819-155-8-201110180-00009 22007046

[23] Reitsma JB Glas AS Rutjes AWS Scholten RJPM Bossuyt PM Zwinderman AH . Bivariate analysis of sensitivity and specificity produces informative summary measures in diagnostic reviews. J Clin Epidemiol. (2005) 58:982–90. doi: 10.1016/j.jclinepi.2005.02.022 16168343

[24] Rutter CM Gatsonis CA . A hierarchical regression approach to meta-analysis of diagnostic test accuracy evaluations. Stat Med. (2001) 20:2865–84. doi: 10.1002/sim.942 11568945

[25] Deeks JJ Macaskill P Irwig L . The performance of tests of publication bias and other sample size effects in systematic reviews of diagnostic test accuracy was assessed. J Clin Epidemiol. (2005) 58:882–93. doi: 10.1016/j.jclinepi.2005.01.018 16085191

[26] Viechtbauer W . Conducting meta-analyses in R with the metafor package. J Stat Softw. (2010) 36:1–48. doi: 10.18637/jss.v036.i03

[27] Zwanenburg A Vallières M Abdalah MA Aerts HJWL Andrearczyk V Apte A . The Image Biomarker Standardization Initiative: standardized quantitative radiomics for high-throughput image-based phenotyping. Radiology. (2020) 295:328–38. doi: 10.1148/radiol.2020191145 32154773 PMC7193906

[28] Tejani AS Klontzas ME Gatti AA Mongan JT Moy L Park SH . Checklist for artificial intelligence in medical imaging (CLAIM): 2024 update. Radiol Artif Intell. (2024) 6:e240300. doi: 10.1148/ryai.240300 38809149 PMC11304031

